# The Potential Benefit by Application of Kinetic Analysis of PET in the Clinical Oncology

**DOI:** 10.5402/2012/349351

**Published:** 2012-12-26

**Authors:** Mustafa Takesh

**Affiliations:** Nuclear Medicine Department, Heidelberg University Hospital, Im Neuenheimer Feld 400, 69120 Heidelberg, Germany

## Abstract

PET is an appropriate method to display the functional activities in target tissue using many types of traces. The visual assessment of PET images plus the semiquantitative parameter (SUV) are the main diagnostic standards considered in identifying the malignant lesion. However, these standards lack occasionally the proper specificity and/or sensitivity. That emphasizes the importance of considering supplemental diagnostic criteria such as the kinetic parameter. The latter gives the way to image the ongoing metabolic processes within the target tissue as well as to identify the alterations occurring at the microscale level before they become observable in the conventional PET-imaging. The importance of kinetic analysis of PET imaging has increased with newly developed PET devices that offer images of good quality and high spatial resolution. In this paper, we highlighted the potential contribution of kinetic analysis in improving the diagnostic accuracy in intracranial tumour, lung tumour, liver tumour, colorectal tumour, bone and soft tissue tumours, and prostate cancer. Moreover, we showed that the appropriate therapy monitoring can be best achieved after considering the kinetic parameters. These promising results indicate that the kinetic analysis of PET imaging may become an essential part in preclinical and clinical molecular imaging as well.

## 1. Introduction

In clinical practice the visual evaluation of PET finding in addition to the semiquantitative parameter (SUV) are the main criteria in delineation of tumor focus. Indeed the uptake measured in the static imaging is a consequence of multiple succeeding miniprocesses that may be exposed by the kinetic analysis of dynamic PET acquisition. That occurs via a sophisticated program depending on complex mathematical relations. 

In the different PET technologies, the ultimate uptake of radiopharmaceutical consists of a set of reaction pathways with corresponding rate coefficients and reverses rate coefficients. The dynamic PET against static PET may demonstrate these reactions after the kinetic analysis, offering the best methodology to understand the uptake mechanism for various clinical and research applications. The background of kinetic analysis of PET imaging depends on suggestion that the target tissue consists of multiple, homogeneous mixed compartments. The resulting rate constants represent the interactions that occur between these compartments including the simple transport and the chemical reforming. 

This technique enables to understand the tracer distribution in the target tissue and to have idea about the metabolic processes occurring at the molecular level, which may be of high clinical value. For example, the knowledge of elementary reactions may contribute in differentiation between the different tissue types, which share a radiopharmaceutical affinity. Furthermore, the therapy via this method can be better and sooner monitored by observing the micro-alterations rather than depending on sole observing of uptake modification. 

The increased glycolysis in the most malignancies makes ^18^F-FDG-PET the most common diagnostic method used in tumor imaging. After intravenous injection, ^18^F-FDG will be transferred across the cell membrane by sodium-independent, facilitative glucose transporters. These transporters are overexpressed in tumor cells [1]. After entering the cell the ^18^F-FDG and glucose will be phosphorylated by hexokinase to ^18^F-FDG-6 phosphate. The fluorine atom in ^18^F-FDG-6-phosphate prevents it to be further metabolized in the glycolytic pathway [2]. That leads to trapping of ^18^F-FDG-6 phosphate and firm continuing accumulation in tumor cells. These microprocesses as well as the possible affecting factors can be demonstrated and assessed quantitatively via kinetic analysis of PET acquisition. 

The increased glycolysis is not the sole key factor in PET imaging. In practice, there are many radiopharmaceuticals differing according to the dominant metabolic alteration in the target tissue. In prostate cancer for example the role of ^18^F-FDG-PET is limited since the glycolysis is very low unless in PCA with high Gleason score. Here the observed overexpression of fatty acid synthesis and the over production of choline kinase was clinically of value by using tracer labeled choline in the PET imaging as alternative choice. The kinetic feature of choline uptake was also reported in similar way to FDG kinetic [3–7].

The performing of dynamic acquisition is an essential condition in accomplishing the kinetic analysis. The period of the dynamic phase varies in relation to the used tracer. In general, this period is required to cover all metabolic processes of the applied tracer and may take up to 1.5 hours. This seems unpractical to be applied routinely to the patients referred for common clinical investigations and minimizes surely the total number of patients may be tested per day. Strauss et al. [8] found that a shortened acquisition protocol may predict the kinetic parameters of the 2-tissue-compartment model in ^18^F-FDG-PET, which means we can theoretically dispense the 1 hour dynamic phase. However there is no more data about the possibility to abbreviate the dynamic phase in other PET applications.

The measurement of input function is an essential part in performing the kinetic analysis. That requires theoretically serial of arterial blood sampling and poses another technical problem we may face in the practice. However, the input function can be retrieved from the image data with good accuracy [9]. So the absence of accurate measurement of the input function may be compensated with drawing a VOI consisting of many ROIs over an arterial vessel in the field of view. 

The choosing of the appropriate model which is supposed to relate to the applied tracer is another important issue should be focused on for a correct kinetic analysis. Two compartment model is a feasible model and used mostly to describe the kinetic features of ^18^F-FDG uptake. 

In this model, we assume that the tissue is combined of two homogeneous mixed interacting compartments. The rate constants *k*_1_ and *k*_2_ refer to, respectively, forward and reverse transport of tracer across the membrane, whereas *k*_3_ and *k*_4_ refer to metabolism and reverse metabolism of the entering tracer respectively, for example phosphorylation and dephosphorylation for ^18^F-FDG. The global influx of the tracer can be retrieved from the previous kinetic parameters according to the formula: influx = (*k*_1_ × *k*_3_)/(*k*_2_ + *k*_3_). Vascular fraction (VB) considers the blood activity within the target area. The unit for rate constants *k*_1_–*k*_4_ is 1/min, whereas VB is a relative measure. Details about the compartment models are described by Burger and Buck [10]. 

In the past few years, the kinetic analysis of PET imaging showed an increasing value in the tumor diagnosis as well as in tumor therapy through providing additional indicative parameters. In therapy monitoring, many authors showed the benefit of kinetic analysis of anticancer drugs after labelling with radionuclide in measuring the specific therapeutic effect [11]. This value brings to light the feasibility of applying the kinetic analysis to the dynamic acquisition which is performed routinely such as in prostate cancer. Here a dynamic phase is performed for better assessing of the prostate bed before the arrival of the eliminated radioactivity in the bladder. This dynamic phase can be further evaluated through kinetic analysis to extract additional parameters may serve for diagnostic purpose. 

Our aim in this paper was to summarize the results of the most published kinetic PET studies using different tracers to demonstrate to which extent the kinetic analysis of PET imaging may play a clinical role in increasing the diagnostic accuracy. 

The additional purpose was to illustrate the potential value of kinetic analysis in the therapy monitoring.

## 2. Kinetic Analysis of PET Imaging in Intracranial Tumours Using Different Tracers

### 2.1. Kinetic of ^18^F-FDG-PET

The value of ^18^F-FDG PET in detecting the brain tumor is limited due to the high rate of physiologic glucose metabolism in the surrounding normal brain tissue. 

To compensate this deficiency, Spence et al. [12] tried in their study on nineteen patients with supratentorial gliomas to investigate whether the acquisition at the late time point may help in distinguishing the glioma from the gray matter. The patients were imaged twice, once from 0 to 90 min and once again from 180 to 480 min after injection. They found visually an improvement in tumor uptake compared with the gray matter as well as in the semi-quantitative assessment. They tried to explain their results via kinetic analysis of the uptake using a 2-compartment, and thereby they were from the first who pointed out the importance of kinetic analysis in brain tumours. They found that *k*_4_ in the tumor tissue is significantly lower than in normal tissue at the late time point. For this reason the degradation of FDG-6-phosphate and thus the elimination of ^18^F-FDG from normal tissue at the delayed time point will be relatively more than from the tumor tissue, which leads in turn to an increase in the tumor contrast. 

Kawai et al. [13] evaluated the kinetic of ^18^F-FDG uptake in 7 patients with histoligically conformed primary central nervous system (CNS) lymphoma. The tumor uptake value was significantly higher than uptake in the normal cortex (in the control patients). That was explained after considering the kinetic data through the significant increase of the phosphorylation in tumor tissue, while the transport rate did not show a significant discrepancy. Similar results were reported by Nishiyama et al. [14]. They found that kinetic analysis of dynamic ^18^F-FDG-PET may help in the diagnosis of the CNS lymphoma and in therapy monitoring as well.

In patients with head and neck cancer, where the FDG-PET is the most applied imaging modality [15], Huang et al. [16] found that the five-parameter kinetic model was the best kinetic model to characterise the FDG metabolism. Anzai et al. [17] revealed in their study on 15 patients with recurrent heads and neck cancers that the considering of the kinetic data in addition of SUVs improve the reliability of tumor detection. 

In 2012, Huang et al. [18] found in their study on forty patient with newly diagnosed nasopharyngeal tumours that the intratumoral heterogeneity of FDG correlates considerably with tumor aggressiveness. That shows the importance of fractal dimension, which is a non-compartmental kinetic parameter to assess the heterogeneity of radiopharmaceutical with further advantage that it is no need of the input function [19, 20]. This may be added to the reasons of kinetic analysis of PET imaging in patient with nasopharyngeal tumors.

### 2.2. Kinetics of Amino Acid PET Tracers

The clinical use of amino acid PET Tracers such as ^11^C-methionine, ^18^F-fluoro-ethyl-tyrosine (FET) and fluorine-18-dihydroxyphenylalanine (^18^F-DOPA) has increased remarkably in brain tumors diagnosis taking into consideration the shortcoming of ^18^F-FDG-PET [21–23]. Chen et al. showed that ^18^F-DOPA PET was superior to ^18^F-FDG-PET in imaging of low-grade tumors and recurrent tumors [24].

About the uptake mechanisms, which is important in explaining the kinetic date, Ishiwata et al. [25] showed that after entering the tumor cell the amino acids get involved in protein synthesis through multiple anabolic and catabolic processes. However, the transport according to Ishiwata et al. is the main event affecting the PET signal and the other processes are far to be demonstrated during the short time of PET acquisition.

In 2007, the kinetic analysis of ^18^F-DOPA uptake in brain tumors was first reported by Schiepers et al. [26] after performing a 75 min dynamic acquisition using compartmental modeling. They included a total of 37 patients with different grading of brain tumors and compared the kinetic data of tumour tissue with that of neighbouring striatum. They found out that ^18^F-DOPA in tumours was only transported without trapping, unlike the case in the striatum. Moreover they found that the form of the uptake curve relates to the tumor grade, which in their opinion may be helpful in distinguishing the high-grade tumors from low-grade tumors.

### 2.3. Kinetics of Hypoxia PET Tracer

PET tracer to image hypoxia such as ^18^F-Fluoromisonidazole (FMISO) was shown to be of value in brain tumors. In 2006 Cher et al. [27] showed in their study on 17 patients that ^18^F-FMISO-PET assesses effectively the hypoxia in gliomas. Moreover they found that this method is valuable in monitoring the targeted hypoxic therapies. Thorwarth et al. [28] described in 2005 an method to quantify the tumor hypoxia in the head and neck tumor based on kinetic analysis of dynamic ^18^F-fluoromisonidazole PET using two compartment model. They found that the assessment of hypoxia depending on kinetic data is more reliable than that derived from static standardized uptake values (SUV). They explained this superiority for the reason that the tissue with intensive hypoxia and necrosis show a low uptake in the static images. 

Thorwarth et al. [29] studied the kinetic of FMISO in 15 patients with advanced stage head and neck cancer prior to radiotherapy by performing 60 min dynamic acquisition. They found high correlation between the kinetic parameters and the therapy outcomes. 

In a study by Shi et al. [30] in patients with advanced head and neck tumors using another hypoxia tracer ^18^F-fluoroazomycin arabinoside (^18^F-FAZA), it was found that kinetic modeling may provide different information from static measurements.

In the framework of developing the kinetic analysis, Hong et al. [31] described in their trial on rat ischemic stroke using micro-PET with [^18^F]fluoromisonidazole a method for more accurate calculation of kinetic parameters. Wang et al. [32] applied mathematical phantom studies to provide a guidance in clinical dynamic FMISO-PET studies.

### 2.4. Kinetics of Somatostatin Receptor-PET Tracer (^68^Ga-DOTA-TOC)

The role of ^68^Ga-DOTA-TOC in the intracranial tumors in particular meningioma was reported by Henze et al. in 2001 [33]. They described very promising results even in the small meningiomas. The kinetic analysis of uptake of ^68^Ga-DOTA-TOC gains importance because thereby the receptor binding can be assessed. Henze et al. [34] described the kinetic model of ^68^Ga-DOTA-TOC in 21 patients with meningiomas. By that they explained the high uptake through high values of vascular fraction (VB) and low values of *k*_2_ and *k*_4_. This model in their opinion provides a sufficient description of the biologic features of meningiomas and may offer the basis for monitoring the radiotherapy. 

### 2.5. Kinetic of PET Tracer Indicative for Cellular Proliferation (Thymidine)

^18^F-fluorothymidine-PET (FLT) has been used in PET tumour imaging as biomarker for cellular proliferation. According to our knowledge, Jacobs et al. [35] were the first who described the kinetic modeling of FLT on 14 patients with glioma of different grades. They found the FLT uptake is fundamentally due to the increased transport. Moreover they found that the contribution of phosphorylation in the uptake increases with the tumour grade. One year later Muzi et al. [36] compared the kinetic results with MR images of blood-brain barrier (BBB) breakdown assessed by gadolinium contrast enhancement. They found that the transport dominates particularly in tumour with BBB breakdown.

The relationship between tumor grading and the kinetic parameters was described by Schiepers et al. [37] in studying the kinetic of FLT in patients with different grade of brain tumour using a three-compartment model. Also they found that the kinetic parameters correlate with the clinical follow up, so the tumour processing may be to some extent estimated in view of the kinetic results.

After specific antiproliferation therapy, Bradbury et al. [38] described in trails on glioma-bearing mice after performing 60 min dynamic FLT-PET a promising role of kinetic analysis in the reliable assessment of cellular proliferation and therapy monitoring. 

Similar results were reported by Schiepers et al. [39]. They pointed out the value of kinetic modeling in the monitoring of bevacizumab and irinotecan therapy in patients with recurrent high-grade glioma. They performed 1 h dynamic FLT-PET study at baseline, after 1 course and at the end of therapy. They showed a significant drop in SUV which was correlated with the influx rate. This correlation in their opinion approves the possibility of response monitoring simply by observing the uptake alterations. However, in a more recently published study, Wardak et al. [40] demonstrated a rather conflicting results. They found that the changes in the single parameters or SUV are not sufficient to the precise evaluation of the therapy and the optimal prediction of therapy outcome required the considering of a set of ^18^F-FLT kinetic parameters. That was in their study on 18 patients with recurrent high-grade glioma after performing a three 1 hour dynamic FLT-PET; at the baseline; 2 and 6 weeks after the treatment. 

## 3. Kinetic Analysis of PET Imaging in Lung Cancer

The diagnostic role of FDG-PET in lung cancer is well known. This role may be extended beyond tumor detection to the discrimination between the different histological types of lung cancer. This further benefit can be achieved using the kinetic analysis of ^18^F-FDG-PET. In 2011, Tsuchida et al. [41] described the importance of kinetic analysis of PET in differentiation between the histological subtypes of lung cancer in their study on 44 patients with histological confirmed lung caner including squamous cell carcinoma, well-differentiated adenocarcinoma and poorly/moderately differentiated adenocarcinoma. They demonstrated that both *k*_1_ and *k*_3_ differ significantly among the 3 groups, with highest values in SCC and lowest in well-differentiated adenocarcinoma. That was attributed to the differences in glucose transporter concentration and hexokinase activity between tumour types. 

Torizuka et al. [42] suggested in their study on 19 patients with non-treated lung cancer after kinetic analysis of dynamic ^18^F-FDG-PET that phosphorylation rate is maybe not crucial for ^18^F-FDG accumulation, given that they found a poor correlation between the *k*_3_ (phosphorylation rate) and SUV.

Regarding the other tracer may be utilized in lung tumor diagnosis, Juhász et al. [43] showed using the kinetic analysis a high transport and metabolism of tryptophan in lung tumours with higher proliferation rates. Accordingly they suggested that (AMT) alpha-methyl-tryptophan-PET may be of clinical value in lung cancer in particular in monitoring of immunopharmacotherapy. Muzi et al. [44] studied the kinetic of fluorothymidine (FLT-PET) using two compartment model with blood sampling in seventeen patients and showed that FLT flux retrieved from the kinetic analysis was correlated with the in vitro measures of tumour proliferation done after surgical resection of tumours. 

The clinical benefit of the kinetic analysis in PET was not only demonstrated in the diagnostic field of lung cancer but also in the therapy. For example the kinetic of the mediation used in the chemotherapy leads to a more accurate assessment of molecular distribution within the tumour tissue. Van Der Veldt and co-authors [45] studied in 2011 the kinetic modeling of docetaxel after labelling with ^11^C in 34 patients with lung cancer. They found that the heterogency in ^11^C-docetaxel kinetics in lung tumours may be an indication of difference of sensitivity to docetaxel. They showed that the tumours with a high influx value had a significantly better response than tumours with a lower influx value. Moreover they revealed that the pretherapy with dexamethason is of significant effect on the kinetic model of docetaxel. 

In the literature there are many similar studies, which exhibit the importance of studying the pharmacokinetic of anticancer drugs after labelling with radionuclide in a better therapy assessment [11]. The same issue was discussed elsewhere in this paper by assessing the fluorouracil kinetic in the colorectal tumours using PET technique.

## 4. Kinetic Analysis of PET Imaging in Liver Tumours

In hepatocellular carcinoma (HCC) imaging, the role of ^18^F-FDG-PET is limited except in patients with poorly differentiated HCC [46]. Yet after using the kinetic analysis of dynamic PET this limitation may be overcome. In 1999, Okazumi et al. [47] pointed out the importance of kinetic modeling in distinguishing the HCC from the normal tissue in their study on a population of 35 patients after performing 60 min dynamic PET studies. They compared the kinetic results with the hexokinase activity in the excised tumour samples and found that *k*_3_ reflects tumour hexokinase activity and with a certain cut off value it is possible to distinguish benign and malignant tumours; furthermore even the degree of differentiation of HCC can be assessed considering the rate constant *k*_3_ and *k*_4_.

Moreover, they found that after therapy the *k*_3_ drop corresponds with the therapy efficacy, for this reason the kinetic analysis seems to offer an extra advantage in the therapy monitoring. 

Choi et al. [48] showed that the state of dietary affects significantly the hepatic FDG kinetics in their study on 10 normal volunteers who underwent two PET studies once after fasting and once again after oral consumption of dextrose. In their study, the fasting was better for the tumour-to-background contrast.

In patients with liver metastases, Messa et al. [49] showed that the parametric images extracted from the kinetic data can contribute in increasing the contrast against the normal liver tissue. Moreover they showed that the higher levels of glucose-6-phosphatase in normal liver tissue compared with liver metastases resulted to higher values of *k*_4_ and thereby an increased elimination of radioactivity from normal tissue. This result mimics the results of Spence et al. [12] in patients with supratentorial gliomas.

About the kinetic study of another tracer may be applied in liver tumour diagnosis, Chen et al. [50] described in 2004 the kinetic model of ^11^C-acetate uptake in HCC using 3 compartment model and pointed out its importance in HCC imaging. They described in another study [51] a non-invasive method of quantification of the sole portal and arterial contribution to the whole blood supply depending on the ^11^C-acetate kinetic model extracted after performing 10 min dynamic PET. They showed that this assessment lead to a better understanding of blood supply mechanism in the liver and may help in the early detection of liver tumour. 

## 5. Kinetic Analysis of PET Imaging in Colorectal Tumours

The role of ^18^F-FDG-PET in detecting the colorectal cancer is well known. However the visual assessment of PET finding besides the semiquantitative analysis may lack the required accuracy. Strauss et al. [52] were among the first who showed the superiority of kinetic analysis of ^18^F-FDG-PET over the visual assessment in colorectal tumours. In 2007, they included 22 patients with colorectal tumor prior surgery who underwent a 60 min dynamic PET studies. They found that the kinetic analysis may help in differentiation of primary colorectal tumours from normal tissue. Moreover they revealed that kinetic analysis of primary tumor may predict the presence of the distant metastases. 

In 2008, Strauss et al. [53] compared the kinetic data with the gene expression (assessed by gene arrays on tissue samples) in 25 patients with colorectal tumors. They found that the kinetic model in particular *k*_1_ was correlated with the expression of the angiogenesis-related genes. In the same topic and more recently Strauss et al. [54] showed in their study on 25 patients that FDG kinetics model in particular the parameters for the transport (*k*_1_) was related with the genes of proliferation, which means that the biological behaviour including angiogenesis and cellular proliferation may be predicted in the light of kinetic data.

The role of kinetic analysis of PET imaging in colorectal tumours goes beyond the improving of diagnostic accuracy to the accurate planning of radiotherapy. In 2009, Buijsen et al. [55] investigated the role of ^18^F-FDG-PET compared with MR in the accurate definition of target volume before the radiotherapy in twenty-six patients with rectal cancer. The patients underwent surgical removal of the tumour within 3 days after RT. The authors found that the PET-based tumour volume was better correlated with the surgical sampling. However, the accurate definition of target volume using PET might be affected by the choosing the threshold level. That was discussed by Ford et al. in their study on patients with head and neck tumours [56]. As possible resolution of this shortcoming, Janssen et al. [57] tried using dynamic PET to develop a method for tumour delineation derived from time-activity curve; they had promising results and suggested further researches to validate the possible superiority of tumour delineation based on dynamic PET analysis. 

The role of kinetic analysis should be also mentioned in the therapy monitoring. In 2003, Bading et al. [58] suggested in their study in rats using ^18^F fluorouracil that the kinetic analysis using compartmental modeling provide very useful quantitative information about 5-FU (fluorouracil) metabolism within the tumour, which may help in predicting the tumour response to 5-FU. This value was pointed out in a more recent study done by Strauss et al. [59]. They assessed using dynamic PET studies the chemotherapeutic effects of the FOLFOX protocol (fluorouracil, folinic acid, oxaliplatin) in mice implanted with a human colorectal cell. They showed that even one therapy may affect the FDG kinetics, which was important to identify the tumours with a rash chemotherapeutic effect.

Dimitrakopoulou-Strauss et al. [60] showed in their study on 28 patients with metastatic colorectal cancer, that the combination of SUV in the static imaging with FD (fractal dimension) retrieved from the kinetic analysis provide the best results in monitoring of chemotherapy. They showed in another study on 25 patients also with metastatic colorectal carcinoma after receiving FOLFOX chemotherapy, that the combination of kinetic parameters provided prognostic aspects in classifying the patients into a short or long survival class [61]. 

## 6. Kinetic Analysis of PET Imaging in Bone and Soft Tissue Tumours

Clinically, it is of special value in case of bone lesion first to exclude the malignancy, because the following procedure is totally different according to the results. The role of ^18^F-FDG-PET in detecting and staging the bone tumors was described in many studies, even the prediction of the nature of bone lesion and discrimination between the different bone lesions can be achieved based on the absolute SUV value [62–64]. Schulte et al. [62] reported an accuracy of 81.7% for malignancy with a cut-off SUV level of 3.0. However, this method faces a major limitation because of the significant overlap between the benign and malignant lesions. To overcome this difficulty, some authors suggested a dual time point acquisitions as a potential procedure to distinguish the malignant lesion from the benign lesions [65].

In this critical topic the importance of kinetic analysis of FDG is emphasized, Dimitrakopoulou-Strauss et al. showed the importance of kinetic analysis in differentiation between benign and malignant bone tumor [66]. They showed in their study on 83 patients with malignant and benign lesions, that the combination of SUVs and the kinetic parameters revealed a sensitivity of 75.86% and specificity of 97.22% in distinguishing the malignant lesion from benign lesion versus sensitivity 54.05% and specificity of 91.30% when depending only on SUV values.

The additional benefit of the kinetic modeling is in identifying some bone lesions such as giant cell tumor (GCT) through illustrating the special kinetic properties. This tumor is characterized with high FDG accumulation in spite of the benign nature. That was attributed to the enhanced vascular fraction and increased ^18^F-FDG transport according to the kinetic study reported by Strauss et al. [67]. The authors showed also a close association of quantitative ^18^F-FDG results and the expression of angiogenesis genes. The knowledge of these special features of GCT is of clinical benefit in avoiding the confusion with other malignant tumors, since the both may demonstrate a high FDG uptake in the static images. 

In this regard, it is worth mentioning that kinetic analysis of ^18^F-FDG-PET in assessing the perfusion and volume of distribution in GCT mimics the analysis of dynamic contrast-enhanced MRI. Here GCT was also shown to have a high vascular distribution and analogous to that in malignant tumors [68]. 

In assessing the soft tissue tumours, ^18^F-FDG-PET faces the same limitation when depending on the sole visual assessment and the semiquantitative evaluation using SUV. Because this assessment may lead to false negative in the low grade malignancy. The clinical benefit of PET kinetic analysis was discussed by Dimitrakopoulou-Strauss et al. [69] in 2001; they included 56 patients with malignant and benign tumors and applied the kinetic analysis after performing a 60 min dynamic acquisition. They showed that the differentiation between the different soft-tissue tumors with different grading was best achieved by using the kinetic parameters in addition to the SUV value. Another study by Okazumi et al. [70] in large patient population confirmed these data. In 2009 they evaluated 117 patients with malignant and benign soft tissue tumors and showed that the kinetic studying of ^18^F-FDG-PET provides important clinical information regarding the histological grading and prognosis. In patients with soft-tissue sarcomas and receiving neoadjuvant chemotherapy Dimitrakopoulou-Strauss et al. [71] pointed out the role of kinetic analysis of PET in early prediction of therapy response. They considered the histopathologic response as reference and found that the combined use of SUV and influx lead to the highest accuracy in predicting therapy response.

## 7. Kinetic Analysis of PET Imaging in Prostate Cancer

As earlier mentioned the clinical use of ^18^F-FDG-PET in PCA diagnostic is limited unless in PCA with high Gleason score. Imaging of tumor lipid metabolism such as choline-PET was introduced clinically as alternative choice for PCA diagnosis, since PCA is characterized by high levels of phospholopid metabolites.

To understand the kinetic model of choline and its clinical applications it may be important to know the molecular pathway of choline in the tumor cells. The first step of choline uptake in tumor cell is the transport across the cellular membrane through three kinds of transport system [72]. The entering choline will be phosphorylated via choline kinase. There are conflicting results about the contribution of choline transport and phosphorylation in the ultimate choline uptake. In the experimental studies it was suggested that the transport is the key factor in the uptake [2, 73]. In another study it was demonstrated that the phosphorylation mediated choline kinase is of significant role in increasing the intracellular trapping however at late time point [74]. Therefore, it is of importance to have a sufficient dynamic period to demonstrate the whole metabolic processes and to reveal truly the metabolic path of choline uptake. 

The first study aimed at this purpose was carried out by Sutinen et al. in 2004 [3]. The researchers assessed the kinetics of the uptake of ^11^C labeled choline in patients with histologically confirmed prostate cancer and benign prostatic hyperplasia after performing a dynamic emission acquisition for 30 min. They showed that the measured SUV is close correlated with kinetic influx constant, which in their opinion support the use of simple SUV in clinical setting. Moreover they showed that a high uptake of ^11^C-choline does not only characterize the prostate tumor but also hyperplastic prostatic tissue.

^11^C labeled acetate is another tracer may be used in diagnosis of prostate cancer [75, 76]. In vitro as well as in vivo studies the acetate uptake was supposed to be related to the fatty acid synthesis [77]. It will be transported by monocarboxylate carriers before getting involving in further metabolic processes. As mentioned previously, ^11^C-acetate can be used in HCC diagnosis; here the kinetic analysis was described by Chen et al. [50]. In 2008, Schiepers et al. [78] had studied for the first time the kinetic of ^11^C-acetate in patients with primary prostate tumor and other with recurrent tumor after performing a 20 min acquisition phase and using standard two tissue model. They found significant differences between primary and recurrent cancer in the transport, influx and distribution volume. Moreover they found a high correlation between ^11^C-acetate uptake in primary tumor and influx rate constant comparable to the results reported by Sutinen et al. in studying the kinetic of ^11^C-choline. That in turn validates the clinical use of simple uptake measurements (SUV) in the routine practice. Ultimately the kinetic analysis of the tracer used in PCA diagnosis was of clinical value and extended the knowledge of the uptake mechanisms in the target tissue. 

## 8. Conclusion

The need of an additional method to compensate the shortcomings of visual and semiquantitative assessment of PET findings has increased in the clinical routine of tumour diagnosis. The kinetic analysis of dynamic PET imaging with a more accurate assessment of changes in tumour metabolism has been shown to be effective in improving the diagnostic accuracy in many different types of cancer. Moreover, the appropriate therapy monitoring can be best achieved after considering the kinetic parameters.

The promising results shown in the literature indicate that the kinetic analysis will witness a great progress in the near future, so it can be applied routinely in tumour diagnosis. Thus the kinetic analysis of PET may become an essential element in the preclinical and clinical molecular imaging as well.

## References

[1] Macheda ML, Rogers S, Best JD (2005). Molecular and cellular regulation of glucose transporter (GLUT) proteins in cancer. *Journal of Cellular Physiology*.

[2] Plathow C, Weber WA (2008). Tumor cell metabolism imaging. *Journal of Nuclear Medicine*.

[3] Sutinen E, Nurmi M, Roivainen A (2004). Kinetics of [^11^C]choline uptake in prostate cancer: a PET stydy. *European Journal of Nuclear Medicine and Molecular Imaging*.

[4] Glunde K, Bhujwalla ZM (2007). Choline kinase alpha in cancer prognosis and treatment. *Lancet Oncology*.

[5] Beheshti M, Langsteger W, Fogelman I (2009). Prostate cancer: role of SPECT and PET in imaging bone metastases. *Seminars in Nuclear Medicine*.

[6] Breeuwsma AJ, Pruim J, Jongen MM (2005). In vivo uptake of [^11^C]choline does not correlate with cell proliferation in human prostate cancer. *European Journal of Nuclear Medicine and Molecular Imaging*.

[7] Zheng QH, Gardner TA, Raikwar S (2004). [^11^C]Choline as a PET biomarker for assessment of prostate cancer tumor models. *Bioorganic and Medicinal Chemistry*.

[8] Strauss LG, Pan L, Cheng C, Haberkorn U, Dimitrakopoulou-Strauss A (2011). Shortened acquisition protocols for the quantitative assessment of the 2-tissue-compartment model using dynamic PET/CT^18^F-FDG studies. *Journal of Nuclear Medicine*.

[9] Ohtake T, Kosaka N, Watanabe T (1991). Noninvasive method to obtain input function for measuring tissue glucose utilization of thoracic and abdominal organs. *Journal of Nuclear Medicine*.

[10] Burger C, Buck A (1997). Requirements and implementation of a flexible kinetic modeling tool. *Journal of Nuclear Medicine*.

[11] Hutchinson OC, Collingridge DR, Barthel H, Price PM, Aboagye EO (2003). Pharmacokinetics of radiolabelled anticancer drugs for positron emission tomography. *Current Pharmaceutical Design*.

[12] Spence AM, Muzi M, Mankoff DA (2004). ^18^F-FDG PET of gliomas at delayed intervals: improved distinction between tumor and normal gray matter. *Journal of Nuclear Medicine*.

[13] Kawai N, Nishiyama Y, Miyake K, Tamiya T, Nagao S (2005). Evaluation of tumor FDG transport and metabolism in primary central nervous system lymphoma using [^18^F]fluorodeoxyglucose (FDG) positron emission tomography (PET) kinetic analysis. *Annals of Nuclear Medicine*.

[14] Nishiyama Y, Yamamoto Y, Monden T (2007). Diagnostic value of kinetic analysis using dynamic FDG PET in immunocompetent patients with primary CNS lymphoma. *European Journal of Nuclear Medicine and Molecular Imaging*.

[15] Schöder H, Yeung HWD (2004). Positron emission imaging of head and neck cancer, including thyroid carcinoma. *Seminars in Nuclear Medicine*.

[16] Huang B, Khong P-L, Kwong DL-W, Hung B, Wong C-S, Wong C-YO (2012). Dynamic PET-CT studies for characterizing nasopharyngeal carcinoma metabolism: comparison of analytical methods. *Nuclear Medicine Communications*.

[17] Anzai Y, Minoshima S, Wolf GT, Wahl RL (1999). Head and neck cancer: detection of recurrence with three-dimensional principal components analysis at dynamic FDG PET. *Radiology*.

[18] Huang B, Chan T, Chan WKS, Khong P-L (2012). Nasopharyngeal carcinoma: investigation of intratumoral heterogeneity with FDG PET/CT. *American Journal of Roentgenology*.

[19] Peitgen HO, Juergens H, Saupe D (1992). *Chaos and Fractals*.

[20] Kleen M, Habler O, Zwissler B, Messmer K (1998). Programs for assessment of spatial heterogeneity of regional organ blood flow. *Computer Methods and Programs in Biomedicine*.

[21] Laverman P, Boerman OC, Corstens FHM, Oyen WJG (2002). Fluorinated amino acids for tumour imaging with positron emission tomography. *European Journal of Nuclear Medicine*.

[22] Weber WA, Wester HJ, Grosu AL (2000). O-(2-[^18^F]fluoroethyl)-L-tyrosine and L-[methyl-^11^C]methionine uptake in brain tumours: initial results of a comparative study. *European Journal of Nuclear Medicine*.

[23] Becherer A, Karanikas G, Szabó M (2003). Brain tumour imaging with PET: a comparison between [^18^F]fluorodopa and [^11^C]methionine. *European Journal of Nuclear Medicine and Molecular Imaging*.

[24] Chen W, Silverman DHS, Delaloye S (2006). ^18^F-FDOPA PET imaging of brain tumors: comparison study with ^18^F-FDG PET and evaluation of diagnostic accuracy. *Journal of Nuclear Medicine*.

[25] Ishiwata K, Kubota K, Murakami M (1993). Re-evaluation of amino acid PET studies: can the protein synthesis rates in brain and tumor tissues be measured in vivo?. *Journal of Nuclear Medicine*.

[26] Schiepers C, Chen W, Cloughesy T, Dahlbom M, Huang SC (2007). ^18^F-FDOPA kinetics in brain tumors. *Journal of Nuclear Medicine*.

[27] Cher LM, Murone C, Lawrentschuk N (2006). Correlation of hypoxic cell fraction and angiogenesis with glucose metabolic rate in gliomas using ^18^F-fluoromisonidazole, ^18^F-FDG PET, and immunohistochemical studies. *Journal of Nuclear Medicine*.

[28] Thorwarth D, Eschmann SM, Paulsen F, Alber M (2005). A kinetic model for dynamic [^18^F]-Fmiso PET data to analyse tumour hypoxia. *Physics in Medicine and Biology*.

[29] Thorwarth D, Eschmann SM, Scheiderbauer J, Paulsen F, Alber M (2005). Kinetic analysis of dynamic ^18^F-fluoromisonidazole PET correlates with radiation treatment outcome in head-and-neck cancer. *BMC Cancer*.

[30] Shi K, Souvatzoglou M, Astner ST (2010). Quantitative assessment of hypoxia kinetic models by a cross-study of dynamic ^18^F-FAZA and ^15^O-H_2_O in patients with head and neck tumors. *Journal of Nuclear Medicine*.

[31] Hong YT, Beech JS, Smith R, Baron JC, Fryer TD (2011). Parametric mapping of 18 Ffluoromisonidazole positron emission tomography using basis functions. *Journal of Cerebral Blood Flow and Metabolism*.

[32] Wang W, Lee NY, Georgi JC (2010). Pharmacokinetic analysis of hypoxia ^18^F-fluoromisonidazole dynamic PET in head and neck cancer. *Journal of Nuclear Medicine*.

[33] Henze M, Schuhmacher J, Hipp P (2001). PET imaging of somatostatin receptors using [68GA]DOTA-D-Phe1-Tyr3-Octreotide: first results in patients with meningiomas. *Journal of Nuclear Medicine*.

[34] Henze M, Dimitrakopoulou-Strauss A, Milker-Zabel S (2005). Characterization of 68Ga-DOTA-D-Phe1-Tyr 3-octreotide kinetics in patients with meningiomas. *Journal of Nuclear Medicine*.

[35] Jacobs AH, Thomas A, Kracht LW (2005). ^18^F-fluoro-L-thymidine and ^11^C-methylmethionine as markers of increased transport and proliferation in brain tumors. *Journal of Nuclear Medicine*.

[36] Muzi M, Spence AM, O’Sullivan F (2006). Kinetic analysis of 3′-deoxy-3′-^18^F-fluorothymidine in patients with gliomas. *Journal of Nuclear Medicine*.

[37] Schiepers C, Chen W, Dahlbom M, Cloughesy T, Hoh CK, Huang SC (2007). ^18^F-fluorothymidine kinetics of malignant brain tumors. *European Journal of Nuclear Medicine and Molecular Imaging*.

[38] Bradbury MS, Hambardzumyan D, Zanzonico PB (2008). Dynamic small-animal PET imaging of tumor proliferation with 3′-deoxy-3′-^18^F-fluorothymidine in a genetically engineered mouse model of high-grade gliomas. *Journal of Nuclear Medicine*.

[39] Schiepers C, Dahlbom M, Chen W (2010). Kinetics of *3ʹ*-deoxy-3*ʹ*-^18^F-fluorothymidine during treatment monitoring of recurrent high-grade glioma. *Journal of Nuclear Medicine*.

[40] Wardak M, Schiepers C, Dahlbom M (2011). Discriminant analysis of ^18^F-fluorothymidine kinetic parameters to predict survival in patients with recurrent high-grade glioma. *Clinical Cancer Research*.

[41] Tsuchida T, Demura Y, Sasaki M (2011). Differentiation of histological subtypes in lung cancer with ^18^F-FDG-PET 3-point imaging and kinetic analysis. *Hellenic Journal of Nuclear Medicine*.

[42] Torizuka T, Zasadny KR, Recker B, Wahl RL (1998). Untreated primary lung and breast cancers: correlation between F-18 FDG kinetic rate constants and findings of in vitro studies. *Radiology*.

[43] Juhász C, Lu X, Jahania MS (2009). Quantification of tryptophan transport and metabolism in lung tumors using PET. *Journal of Nuclear Medicine*.

[44] Muzi M, Vesselle H, Grierson JR (2005). Kinetic analysis of 3′-deoxy-3′-fluorothymidine PET studies: validation studies in patients with lung cancer. *Journal of Nuclear Medicine*.

[45] Van Der Veldt AAM, Lubberink M, Greuter HN (2011). Absolute quantification of [^11^C]docetaxel kinetics in lung cancer patients using positron emission tomography. *Clinical Cancer Research*.

[46] Trojan J, Schroeder O, Raedle J (1999). Fluorine-18 FDG positron emission tomography for imaging of hepatocellular carcinoma. *American Journal of Gastroenterology*.

[47] Okazumi S, Isono K, Enomoto K (1992). Evaluation of liver tumors using fluorine-18-fluorodeoxyglucose PET: characterization of tumor and assessment of effect of treatment. *Journal of Nuclear Medicine*.

[48] Choi Y, Hawkins RA, Huang SC (1994). Evaluation of the effect of glucose ingestion and kinetic model configurations of FDG in the normal liver. *Journal of Nuclear Medicine*.

[49] Messa C, Choi Y, Hoh CK (1992). Quantification of glucose utilization in liver metastases: parametric imaging of FDG uptake with PET. *Journal of Computer Assisted Tomography*.

[50] Chen S, Ho C, Feng D, Chi Z (2004). Tracer kinetic modeling of ^11^C-acetate applied in the liver with positron emission tomography. *IEEE Transactions on Medical Imaging*.

[51] Chen S, Feng D (2004). Noninvasive quantification of the differential portal and arterial contribution to the liver blood supply front PET measurements using the ^11^C-acetate kinetic model. *IEEE Transactions on Biomedical Engineering*.

[52] Strauss LG, Klippel S, Pan L, Schönleben K, Haberkorn U, Dimitrakopoulou-Strauss A (2007). Assessment of quantitative FDG PET data in primary colorectal tumours: which parameters are important with respect to tumour detection?. *European Journal of Nuclear Medicine and Molecular Imaging*.

[53] Strauss LG, Koczan D, Klippel S (2008). Impact of angiogenesis-related gene expression on the tracer kinetics of ^18^F-FDG in colorectal tumors. *Journal of Nuclear Medicine*.

[54] Strauss LG, Koczan D, Klippel S (2011). Impact of cell-proliferation-associated gene expression on 2-deoxy-2-[^18^F]fluoro-D-glucose (FDG) kinetics as measured by dynamic positron emission tomography (dPET) in Colorectal Tumors. *Molecular Imaging and Biology*.

[55] Buijsen J, Van Den Bogaard J, Janssen MHM (2011). FDG-PET provides the best correlation with the tumor specimen compared to MRI and CT in rectal cancer. *Radiotherapy and Oncology*.

[56] Ford EC, Kinahan PE, Hanlon L (2006). Tumor delineation using PET in head and neck cancers: threshold contouring and lesion volumes. *Medical Physics*.

[57] Janssen MHM, Aerts HJWL, Öllers MC (2009). Tumor delineation based on time-activity curve differences assessed with dynamic fluorodeoxyglucose positron emission tomography-computed tomography in rectal cancer patients. *International Journal of Radiation Oncology Biology Physics*.

[58] Bading JR, Yoo PB, Fissekis JD, Alauddin MM, D’Argenio DZ, Conti PS (2003). Kinetic modeling of 5-fluorouracil anabolism in colorectal adenocarcinoma: a positron emission tomography study in rats. *Cancer Research*.

[59] Strauss LG, Hoffend J, Koczan D, Pan L, Haberkorn U, Dimitrakopoulou-Strauss A (2009). Early effects of FOLFOX treatment of colorectal tumour in an animal model: assessment of changes in gene expression and FDG kinetics. *European Journal of Nuclear Medicine and Molecular Imaging*.

[60] Dimitrakopoulou-Strauss A, Strauss LG, Rudi J (2003). PET-FDG as predictor of therapy response in patients with colorectal carcinoma. *Quarterly Journal of Nuclear Medicine*.

[61] Dimitrakopoulou-Strauss A, Strauss LG, Burger C (2004). Prognostic aspects of ^18^F-FDG PET kinetics in patients with metastatic colorectal carcinoma receiving FOLFOX chemotherapy. *Journal of Nuclear Medicine*.

[62] Schulte M, Brecht-Krauss D, Heymer B (2000). Grading of tumors and tumorlike lesions of bone: evaluation by FDG PET. *Journal of Nuclear Medicine*.

[63] Kole AC, Nieweg OE, Hoekstra HJ, Van Horn JR, Koops HS, Vaalburg W (1998). Fluorine-18-fluorodeoxyglucose assessment of glucose metabolism in bone tumors. *Journal of Nuclear Medicine*.

[64] Wu H, Dimitrakopoulou-Strauss A, Heichel TO (2001). Quantitative evaluation of skeletal tumours with dynamic FDG PET: SUV in comparison to Patlak analysis. *European Journal of Nuclear Medicine*.

[65] Tian R, Su M, Tian Y (2009). Dual-time point PET/CT with F-18 FDG for the differentiation of malignant and benign bone lesions. *Skeletal Radiology*.

[66] Dimitrakopoulou-Strauss A, Strauss LG, Heichel T (2002). The role of quantitative ^18^F-FDG PET studies for the differentiation of malignant and benign bone lesions. *Journal of Nuclear Medicine*.

[67] Strauss LG, Dimitrakopoulou-Strauss A, Koczan D (2004). ^18^F-FDG kinetics and gene expression in giant cell tumors. *Journal of Nuclear Medicine*.

[68] Kawakami Y, Kunisada T, Sugihara S (2007). New approach for assessing vascular distribution within bone tumors using dynamic contrast-enhanced MRI. *Journal of Cancer Research and Clinical Oncology*.

[69] Dimitrakopoulou-Strauss A, Strauss LG, Schwarzbach M (2001). Dynamic PET ^18^F-FDG studies in patients with primary and recurrent soft-tissue sarcomas: impact on diagnosis and correlation with grading. *Journal of Nuclear Medicine*.

[70] Okazumi S, Dimitrakopoulou-Strauss A, Schwarzbach MHM, Strauss LG (2009). Quantitative, dynamic ^18^F-FDG-PET for the evaluation of soft tissue sarcomas: relation to differential diagnosis, tumor grading and prediction of prognosis. *Hellenic Journal of Nuclear Medicine*.

[71] Dimitrakopoulou-Strauss A, Strauss LG, Egerer G (2010). Impact of dynamic ^18^F-FDG PET on the early prediction of therapy outcome in patients with high-risk soft-tissue sarcomas after neoadjuvant chemotherapy: a feasibility study. *Journal of Nuclear Medicine*.

[72] Michel V, Yuan Z, Ramsubir S, Bakovic M (2006). Choline transport for phospholipid synthesis. *Experimental Biology and Medicine*.

[73] Bansal A, Shuyan W, Hara T, Harris RA, DeGrado TR (2008). Biodisposition and metabolism of [^18^F]fluorocholine in 9L glioma cells and 9L glioma-bearing fisher rats. *European Journal of Nuclear Medicine and Molecular Imaging*.

[74] Henriksen G, Herz M, Hauser A, Schwaiger M, Wester HJ (2004). Synthesis and preclinical evaluation of the choline transport tracer deshydroxy-[^18^F]fluorocholine ([^18^F]dOC). *Nuclear Medicine and Biology*.

[75] Shreve P, Chiao PC, Humes HD, Schwaiger M, Gross MD (1995). Carbon-11-acetate PET imaging in renal disease. *Journal of Nuclear Medicine*.

[76] Kotzerke J, Volkmer BG, Neumaier B, Gschwend JE, Hautmann RE, Reske SN (2002). Carbon-11 acetate positron emission tomography can detect local recurrence of prostate cancer. *European Journal of Nuclear Medicine*.

[77] Vavere AL, Kridel SJ, Wheeler FB, Lewis JS (2008). 1-^11^C-acetate as a PET radiopharmaceutical for imaging fatty acid synthase expression in prostate cancer. *Journal of Nuclear Medicine*.

[78] Schiepers C, Hoh CK, Nuyts J (2008). 1-^11^C-acetate kinetics of prostate cancer. *Journal of Nuclear Medicine*.

